# High-Level Systemic Expression of Conserved Influenza Epitope in Plants on the Surface of Rod-Shaped Chimeric Particles

**DOI:** 10.3390/v6041789

**Published:** 2014-04-21

**Authors:** Natalia V. Petukhova, Tatiana V. Gasanova, Peter A. Ivanov, Joseph G. Atabekov

**Affiliations:** 1 Department of Virology, Lomonosov Moscow State University, Moscow 119991, Russia; E-Mails: petukhovanv@genebee.msu.ru (N.V.P.); tv.gasanova@genebee.msu.ru (T.V.G.); atabekov@genebee.msu.ru (J.G.A.); 2 Belozersky Institute of Physico-Chemical Biology, Lomonosov Moscow State University, Moscow 119991, Russia

**Keywords:** influenza virus, M2e, plant, tobacco mosaic virus, systemic expression, coat protein fusion

## Abstract

Recombinant viruses based on the cDNA copy of the tobacco mosaic virus (TMV) genome carrying different versions of the conserved M2e epitope from influenza virus A cloned into the coat protein (CP) gene were obtained and partially characterized by our group previously; cysteines in the human consensus M2e sequence were changed to serine residues. This work intends to show some biological properties of these viruses following plant infections. Agroinfiltration experiments on *Nicotiana benthamiana* confirmed the efficient systemic expression of M2e peptides, and two point amino acid substitutions in recombinant CPs significantly influenced the symptoms and development of viral infections. Joint expression of RNA interference suppressor protein p19 from tomato bushy stunt virus (TBSV) did not affect the accumulation of CP-M2e-ser recombinant protein in non-inoculated leaves. RT-PCR analysis of RNA isolated from either infected leaves or purified TMV-M2e particles proved the genetic stability of TMV‑based viral vectors. Immunoelectron microscopy of crude plant extracts demonstrated that foreign epitopes are located on the surface of chimeric virions. The rod‑shaped geometry of plant-produced M2e epitopes is different from the icosahedral or helical filamentous arrangement of M2e antigens on the carrier virus-like particles (VLP) described earlier. Thereby, we created a simple and efficient system that employs agrobacteria and plant viral vectors in order to produce a candidate broad*-*spectrum flu vaccine*.*

## 1. Introduction

Influenza A is one of the widespread human and animal viruses. Most of the commercial vaccines contain hemagglutinin and neuraminidase as the protective antigens. Those glycoproteins are highly immunogenic, but considerably variable, as well. At the moment, intensive studies are directed towards creating a broad-spectrum vaccine against influenza that contains conserved viral antigens. Matrix protein M2 is a promising candidate for such a vaccine. Tetramers of M2 form ionic channels of virions; one particle contains 14–68 copies of this protein. It was discovered that the N-terminal part is located on the surface of viral particles. This peptide consists of 23 amino acid residues and was named M2e (external), its sequence remains conserved among different influenza isolates known since 1933 [1].

The immunogenic and protective properties of the influenza M2e epitope have been studied for more than 10 years [2,3,4]. This antigen was previously expressed in *E. coli* as a fusion with HBcAg from human hepatitis virus B [5], TLR5 ligand flagellin [6], coat proteins of woodchuck hepatitis virus [7], papaya mosaic virus (PapMV) [8] and phage Qbeta [9]. Recently some examples of plant-based expression were reported [10,11]; the authors used L1 protein from human papillomavirus HPV-16 or HBcAg as a carriers and viral vectors based on cowpea mosaic virus (CPMV) or potato virus X (PVX) genomes, respectively, but expression levels were low and plant infections limited to inoculated leaves.

This investigation was aimed at studying biological properties of two recombinant viruses created previously for the presentation of the M2e epitope using a cDNA copy of the tobacco mosaic virus (TMV-U1) [12] genome. Chimeric particles can be isolated from infected plants, and improved immunogenicity of the epitope that is repeated in many copies (up to 2000 times per one particle) was shown [13].

## 2. Results and Discussion

### 2.1. Symptoms and Development of Infections Driven by Recombinant TMV-Based Viruses

**Figure 1:**
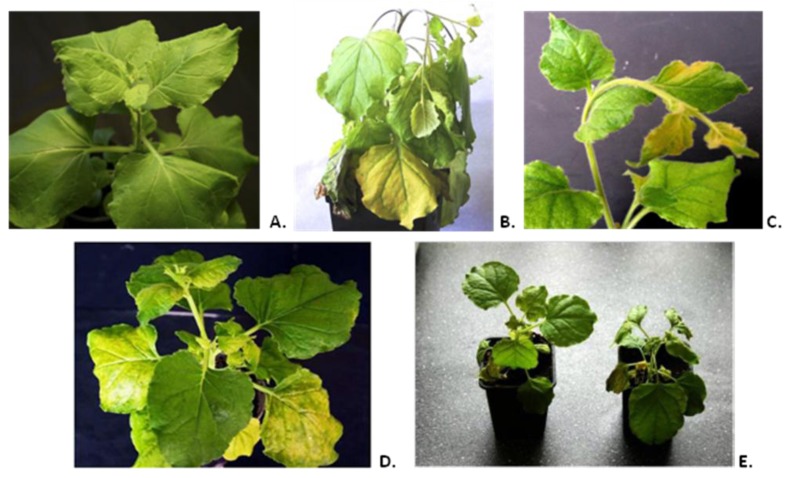
(**A**) Mock-inoculated plant (14 days post inoculation (d.p.i.)). (**B**) Systemic symptoms of the *Nicotiana benthamiana* plant infected by tobacco mosaic virus wild-type (TMV-wt) (14 d.p.i.). (**C**) Systemic symptoms of viral infection on *Nicotiana benthamiana* caused by TMV-M2e-cys (14 d.p.i.). (**D**) Systemic symptoms of viral infection on *Nicotiana benthamiana* caused by TMV-M2e-ser (14 d.p.i.). (**E**) Comparison of systemic symptoms of *Nicotiana benthamiana* plants infected with TMV-M2e-cys (**left**) and TMV-M2e-ser (**right**) at 14 d.p.i.

Cloning of different versions of the human consensus [14] influenza M2e epitope into the open reading frame (ORF) of the CP gene of tobacco mosaic virus (TMV) strain U1 [12] between 155 and 156 codons was described in detail previously [13]. Cysteine codons in the sequence of heterologous peptide were substituted by codons for serine. TMV-M2e-cys and TMV-M2e-ser constructs were transformed into *Agrobacterium tumefaciens*. Infiltrations were performed using two leaves (10–15 sm, third level from the top) of each *Nicotiana benthamiana* plant (three independent experiments, at least ten plants each). An *Agrobacterium*-mediated cDNA copy of TMV wild-type (TMV-wt-agro) served as a positive control. The first symptoms of TMV-wt-agro infection usually appeared 7–8 days post inoculation (d.p.i.) and included typical curling and necrotic lesions of non-inoculated leaves together with the flexion of the upper part of the stem. TMV-M2e-cys infection caused symptoms similar to TMV-wt-agro with additional yellow systemic chlorosis that became visible after 14 d.p.i. The spread of TMV-M2e-ser virus was faster (10 d.p.i.) compared with TMV-M2e-cys infection. Necrotic lesions were absent, and the symptoms (pronounced yellow systemic chlorosis) were different either from the virus with cysteine substitutions or from the wild-type control (Figure 1). 

**Figure 2:**
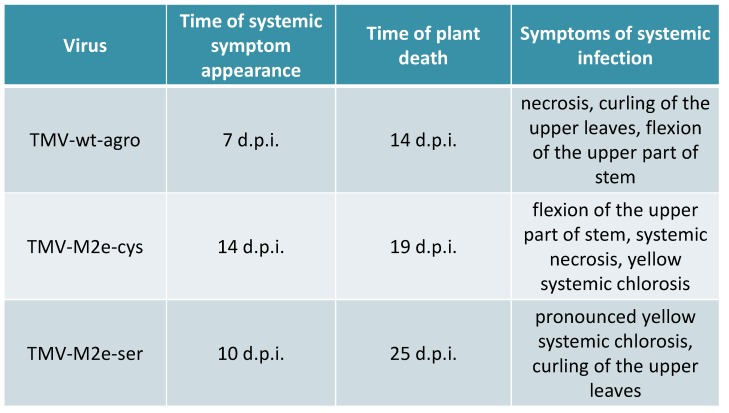
The comparison of symptoms and the development of *Nicotiana benthamiana* infections caused by agroinfiltration of binary vectors coding for TMV-wt and TMV-based recombinant viruses.

All these data are summarized in Figure 2. It should be noted that mixed agroinfiltrations together with bacterial culture expressing the p19 gene led to the faster development of infections: for TMV-M2e-cys, the first symptoms appeared after 11 d.p.i.; for TMV-M2e-ser after 8 d.p.i.

**Figure 3:**
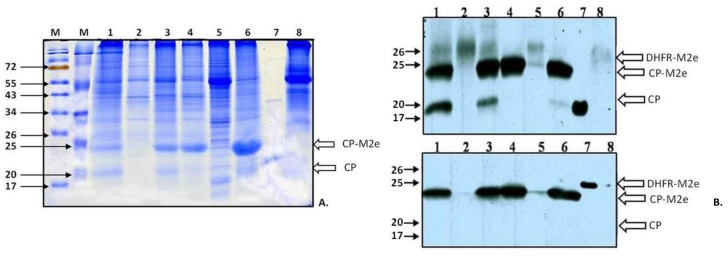
Analysis of the accumulation of TMV-M2e-cys and TMV-M2e-ser CPs in systemically infected plants with or without the expression of p19 from tomato bushy stunt virus (TBSV) (11 d.p.i.). The expression of the p19 protein was only in inoculated leaves. (**A**) SDS-PAGE with Coomassie staining of plant extracts. M, molecular weight markers; the positions of molecular weight markers in kDa are indicated by arrows. **1**: TMV-M2e-cys; the extract of systemic leaves with symptoms, the expression with p19, 1.7 mg of plant tissue; **2**: TMV-M2e-cys; the extract of systemic leaves without symptoms, the expression with p19, 1.7 mg of plant tissue; **3**: TMV-M2e-ser; the extract of systemic leaves with symptoms, the expression with p19, 2 mg of plant tissue; **4**: TMV-M2e-ser; the extract of systemic leaves without symptoms, the expression with p19, 2.2 mg of plant tissue; **5**: TMV-M2e-cys; the extract of systemic leaves with symptoms, 1.5 mg of plant tissue; **6**: TMV-M2e-ser; the extract of systemic leaves with symptoms, 1.6 mg of plant tissue; **7**: positive control CP TMV-wt; **8**: negative control extract of non-infected plant *Nicotiana benthamiana*, 2 mg of plant tissue. (**B**) (**top**) Western blotting with antiserum (AS) to CP TMV; (**bottom**) western blotting with antiserum (AS) to dihydrofolate reductase-M2e (DHFR-M2e) [13] . **1**: TMV-M2e-cys; the extract of systemic leaves with symptoms, the expression with p19, 0.43 mg of plant tissue; **2**: TMV-M2e-cys; the extract of systemic leaves without symptoms, the expression with p19, 1.7 mg of plant tissue; **3**: TMV-M2e-ser; extract of systemic leaves with symptoms, the expression with p19, 0.5 mg of plant tissue; **4**: TMV-M2e-ser; the extract of systemic leaves without symptoms, the expression with p19, 0.55 mg of plant tissue; **5**: TMV-M2e-cys; the extract of systemic leaves with symptoms, 0.75 mg of plant tissue; **6**: TMV-M2e-ser; the extract of systemic leaves with symptoms, 0.16 mg of plant tissue; **7**: positive control, 0.1 µg (CP TMV-wt or DHFR-M2e); **8**: negative control extract of non-infected *Nicotiana benthamiana*, 1.5 mg of plant tissue. White arrows indicate the zones corresponded to DHFR-M2e, CP-M2e and CP proteins.

It is supposed that TMV infection in different *Nicotiana* plants is characterized by very fast systemic spread: within three days after primary inoculation, virus enters the vascular tissue and moves to the upper young leaves, causing visible systemic symptoms. Then it “descends” to the lower and inoculated leaves, so the accumulation of significant amounts of coat protein in these leaves is delayed [15]. Our experiments with recombinant TMV-based viruses confirmed that point of view: Coomassie staining of soluble proteins from inoculated leaves taken 5–8 d.p.i. did not reveal virus‑specific proteins for TMV-M2e-cys; in the case of TMV-wt, only a weak band close to the 20-kDa marker was visible (data not shown). The analysis of systemic leaves (14 d.p.i.) showed that two proteins with electrophoretic mobilities of 20 and 24 kDa were absent in the negative control (Figure 3A).

### 2.2. Analysis of Expression of Modified Coat Proteins

Western blotting of soluble proteins from plant extracts using mouse antiserum (AS) against the M2e epitope [13] or rabbit AS against TMV-wt coat protein (CP) proved that the major 24-kDa protein interacts with both polyclonal antisera and the minor 20-kDa protein interacts only with CP-specific AS (Figure 3B). The mobility of this protein was similar to mobility of the wild-type TMV coat protein (Figure 3A). Taken together, these data allow us to assume that the 20-kDa band corresponds to the shortened version of CP-M2e fusion losing the M2e epitope. Thus, it is possible to conclude that recombinant TMV-M2e-cys and TMV-M2e-ser viruses carrying the conserved influenza M2e epitope are capable of replication, cell-to-cell and long-distance movement in *Nicotiana benthamiana* plants. The accumulation of TMV-M2e-ser was significantly more efficient than TMV-M2e-cys (Figure 3A, Lanes 1, 6). In some experiments, additional *Agrobacterium* carrying the p19 gene from tomato bushy stunt virus (TBSV) [16] was mixed with bacterial culture coding for one of the viral vectors as a putative booster of the expression of the foreign peptide. This protein is a well-known suppressor of RNA interference in plants, so this kind of infection allowed for the investigation of the influence of post‑transcriptional gene silencing (PTGS) to the spread and accumulation of recombinant viruses, as well. Joint expression of silencing suppressor p19 from TBSV influenced the development of TMV‑M2e-cys and TMV-M2e-ser infections (faster symptoms) and the accumulation of CP-M2e-cys (Figure 3A, Lanes 1, 2, 5), but unexpectedly, did not increase the amount of full-length CP-M2e-ser fusion protein in systemic leaves (Figure 3A, Lanes 3, 6). The analysis of the proteins from systemic leaves of plants with long-term infections (more than 20 days) demonstrated that the accumulation level of the 24-kDa protein is decreasing (the 24/20 kDa ratio is 50/50 or less), and in the leaves with large necrotic lesions, a shortened version of the CP-M2e protein predominates (data not shown).

### 2.3. Genetic Stability of Recombinant Viral Genomes

**Figure 4:**
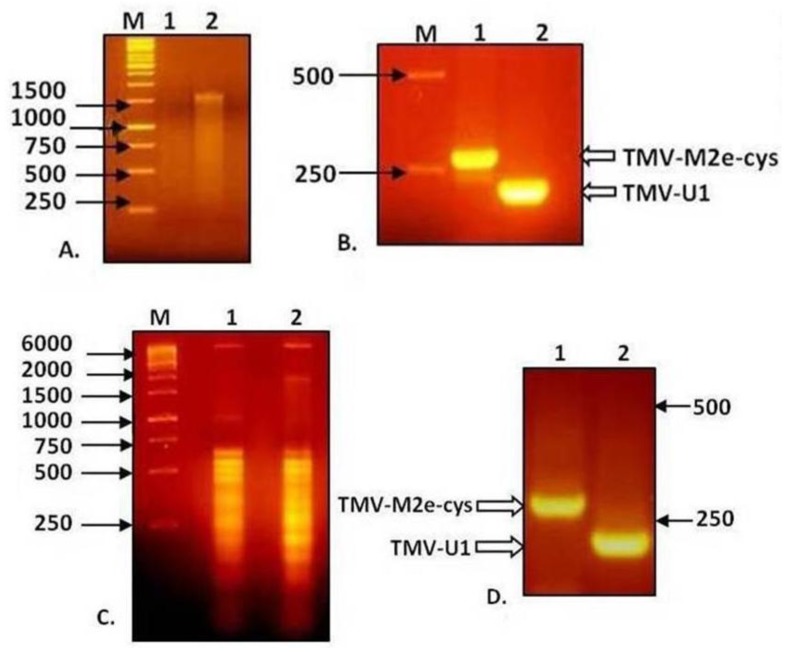
Analysis of RNA of TMV-M2e and RT-PCR products in 2% agarose gel. (**A**) RNA isolation from viral particles preparations. (**B**) Analysis of RT-PCR products derived from the genomic RNA of viral preparations. (**C**) Total RNA isolation from infected plant tissue. (**D**) Analysis of RT-PCR products derived from total RNA of infected plant tissue. **1**: TMV-M2e-cys; **2**: TMV-wt.

Total RNA isolated from non-inoculated leaves with visible symptoms of TMV-M2e-cys and TMV-M2e-ser infections was used for RT-PCR analysis with primers flanking the M2e inserts, as well as RNA extracted from purified viral particles. Electrophoresis in 2% agarose gel showed that the difference in mobility of PCR fragments from wild-type virus and TMV-M2e-cys corresponds with the putative size of the insert (Figure 4).

Similar results were obtained for TMV-M2e-ser (data not shown). All the fragments were purified, and direct sequencing did not reveal any mutations. These data indicate the genetic stability of both recombinant viruses without reversion to the wild-type, so one may suppose that the shortened 20-kDa version of the CP-M2e protein appears because of posttranslational processing, for example proteolytic cleavage of the foreign peptide either before and/or after assembly of chimeric particles.

### 2.4. Immunogold Electron Microscopy of Chimeric Particles in Plant Extracts

**Figure 5:**
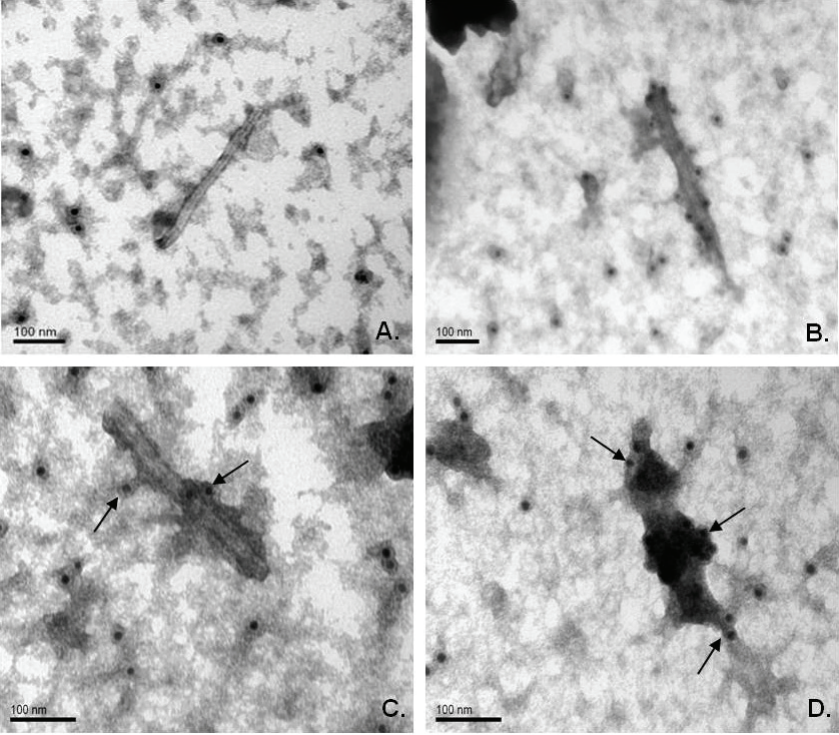
Immunogold labeling of chimeric viral particles in crude plant extracts. (**A**) TMV-wt-agro (wild-type); (**B**, **C**) TMV-M2e-cys; (**D**) TMV-M2e-ser. Primary antibodies, against DHFR-M2e; secondary (anti-mouse) antibodies, conjugated with gold particles (12 nm). Magnifications: **A**, **B**: 200,000; **C**, **D**: 300,000. Scale bars are indicated.

Electronic microscopy of extracts from infected plants revealed rigid rod-shaped particles, which are similar in morphology to wild-type virions. Immunogold labeling clearly demonstrated that the M2e epitope is exposed on the surface of chimeric particles formed by both viral vectors. Crude extracts from symptomatic systemic leaves were treated with primary mouse antiserum against the M2e peptide and secondary antibodies conjugated with gold particles (12 nm). Microscopy demonstrated that chimeric virions have more electron density than TMV wild-type virions, and gold particles were associated with the putative virion-antibody complex. Moreover, it was detected that virions of TMV‑M2e-ser bind many more gold particles; they also bind more uranyl acetate on the sides of the antigen-antibody complexes than TMV-M2e-cys virions (Figure 5).

It might be assumed that TMV-M2e-ser particles have larger amount of M2e epitopes exposed on their surface.

## 3. Experimental Section

### 3.1. Cloning of TMV-wt-agro Construct

Two vectors, pTMV-5' and pA4083, described previously [13], were used to clone the binary vector, pTMV-wt, that contained a full cDNA copy of the TMV-U1 genome. pTMV-wt-agro was generated using the *BamHI/SalI* sites of basic subclones.

### 3.2. Agrobacterium Transformation and Plant Inoculations

Binary viral vectors pTMV-M2e-cys and pTMV-M2e-ser [13] were used for the transformation of *Agrobacterium tumefaciens* strain GV 3101 with the following procedure. Agrobacterial cells were mixed with plasmid and incubated in ice for 30 min. Then, the mixture was heated at 37 °C for 5 min. After heat treatment, 2 mL of liquid nutrient medium LB were added, and the agrobacteria cultures were grown for 2 hours at 28 °C. Then, transformed agrobacterial cells were transferred to solid agar medium with the required antibiotics (rifampicin 50 µg/mL, gentamicin 25 µg/mL, kanamycin 50 µg/mL) for colony growth.

Agrobacterial cultures were grown to the stationary phase from solid media plates, then gently mixed (~5000 × g) at 4–10 °C for 5 min to pellet, and the medium was discarded. Cells were resuspended in buffer for agroinoculation (10 mM 2-(*N*-morpholino)ethanesulfonic acid (MES) pH 5.6, 10 mM MgS0_4_) to an OD_600_ of 0.3. Agroinfiltration was performed on 2–3-level (from the top) leaves that were 8–10 sm across at their widest point. The back side of the leaf was pricked with a razor blade or small pipette tip and gently pressure infiltrated, with the wound against a counter pressure (provided by a finger), with the agrobacteria mixture using a 1- or 2-mL syringe.

### 3.3. Protein Extraction and Coomassie Staining

For protein analysis, infected leaves were homogenized in 3–4 volumes of 50 mM borate buffer (pH 9.0). Proteins remaining in the supernatant after centrifugation (10,000 × g for 5 min) were extracted in 4×SDS protein extraction buffer (60% (v/v) glycerol, 20% (v/v) β-mercaptoethanol, 10% (w/v) sodium dodecyl sulfate (SDS), 0.1% (w/v) bromophenol blue, 250 mМ Tris-HCl, pH 6.8) by heating at 95 °C for 15 min. Proteins were resolved by 15% (w/v) SDS-PAGE at a constant current (15 mA) followed by staining with Coomassie Brilliant Blue.

### 3.4. Western Blotting

Proteins were separated by reducing SDS-PAGE on 15% (w/v) acrylamide gels and electro‑transferred overnight at a constant current (30 mA) to a PVDF membrane (Hybond P, Amersham, The Netherlands). Subsequently, the membrane was blocked with Tris-buffered saline (TBS)-Tween 20 (T) (150 mM NaCl, 10 mM Tris-HCl, 0.1% (v/v) Tween 20, pH 8.0) containing 5% (w/v) nonfat dry milk (“Difco”, Detroit, MI, USA) at room temperature for 1 h and then incubated with either rabbit anti-TMV CP (Department of Virology, Lomonosov Moscow State University, Moscow, Russia, obtained after three intramuscular immunizations, 500 µg of protein per injection, two-week intervals; dilution 1:5000) or mouse anti-DHFR-M2e (dilution 1:15,000) antibodies [13] in 2.5% (w/v) milk/TBS-T for 1 h. After washing with TBS-T, the membrane was incubated for 1 h with anti-rabbit or anti-mouse horseradish peroxidase (HRP)-conjugated secondary antibodies (Sigma A6154 and A4416, respectively; dilution 1:15,000). Signals were generated by chemiluminescence and documented on Hyperfilm (ECL detection system, Amersham, The Netherlands).

### 3.5. RNA Isolation

Preparations of virus particles in 10 mM Tris-HCl, 0.005 M EDTA pH 8.0 [13] were mixed with 10% (w/v) SDS (1/20 volume to a final concentration of 0.5% (w/v)), 1 volume of buffer-saturated with phenol ( 10 mM Tris-HCl, pH 8.0) and 0.25 volumes of chloroform. After centrifugation (13,000 × g, 20 min, 4 °С), RNA from the water phase was precipitated by the addition of three volumes of 96% ethanol and 0.1 volumes of 3 M sodium acetate (pH 5.0). The RNA pellet was resuspended in tri-distilled water.

For total RNA isolation, approximately 1 g of systemically infected leaf tissue, was ground thoroughly in liquid nitrogen and mixed with 6 mL of RNA extraction buffer (200 mM Tris-HCl (pH 9.0), 25 mM EDTA, 1.0% (w/v) SDS) and an equal volume of phenol-chloroform (50:50) followed by vigorous vortexing and a centrifugation step (13,000 × g, 20 min, 4 °С). The upper aqueous phase was collected, and then 3 mL of RNA extraction buffer and an equal volume of phenol-chloroform (50:50) were added to residual phases, mixed and centrifugated (13,000 × g, 15 min, 4 °С). All combined aqueous phases were extracted one more time with an equal volume of phenol-chloroform and three volumes of 96% ethanol, and 0.1 volumes of 3 M sodium acetate (pH 5.0) were added to the water phase and incubated at −70 °C for 2 hours. After centrifugation (13,000 × g, 20 min, 4 °С), the pellet was dissolved in 1 mL of tri-distilled water, then combined with 1/3 volume of 8 M LiCl for a 2 M final concentration and incubated at 4 °C overnight to precipitate the RNA. The pellet obtained after centrifugation (13,000 × g, 20 min, 4 °С) was washed with 80% ethanol and resuspended in tri-distilled water. It should be noted that RNA was isolated at the same time as proteins (Section 3.3 and Section 3.4) were tested.

### 3.6. RT-PCR

To synthesize a first strand cDNA, the purified RNA was mixed with 15 pmol of specific primer Apa-m (5'-tgggcccctaccgggggtaa-3', 6376–6395 nt in TMV U1 genome sequence), and annealing at 75 °С for 5 min was performed. Then, the buffer for reverse transcription (RT) (Fermentas), dNTPs to a 0.5 mM final concentration, 2.5 mM MgCl_2_, 20 units of RNаse Inhibitor (Fermentas) and 200 units of Mu-MLV (Fermentas) were added and incubated for 5 minutes at 28 °С. Reverse transcription was performed at 42 °С for two hours. One tenth of the RT reaction mixture was used as a template for following PCR analysis. The cDNA was amplified during 30 PCR cycles (94 °C for 30 s, 52 °C for 30 s and 72 °C for 60 s) with the CP U1 HindIII-m (5'-actgaagcttcgcaccacgtgtgaattacggacacaat-3', 6220–6244 nt in TMV U1 genome sequence) and the CP U1 PstI-p (5'-actgctgcaggagtagacgacgcaacggtggccata-3', 6052–6077 nt in TMV U1 genome sequence) primers. RT-PCR products were analyzed by 2% (w/v) agarose gel electrophoresis and sequenced. DNA sequencing was performed using the reagent-kit, ABI PRISM^®^ BigDye™ Terminator v. 3.1, with the following analysis of the reaction products on an automatic Applied Biosystems 3730 DNA Analyzer. Sequence Scanner version 1.0 software [17] was used for visualization of data.

### 3.7. Immunogold Labeling

Probes for electron microscopy were prepared using the standard method of negative staining with the 2% (w/v) uranyl acetate solution (pH 6.0) for 2 minutes and examined using a JEOL JEM-1011 electron microscope with Gatan ES 500W Erlangshen digital camera and Digital Micrograph 1.85 software [18].

Plant extracts at concentrations of 30–100 µg/mL were applied to 200 mesh carbon-coated copper electron microscopy (EM) grids (01700F Ted Pella, Redding, CA, USA) and left to settle for 1–2 min. Then, the grids were incubated with 1% (w/v) bovine serum albumin (BSA) solution in 1× PBS for 20 minutes followed by three washes with 1× PBS for 15 min. Thereafter, mouse anti-DHFR-M2e serum [13] (diluted 1:500 in 1× PBS) was added and incubated for 1 hour. After five washes with PBS buffer, the grids were incubated for 40 min with secondary anti-mouse antibodies conjugated with 12-nm gold particles (Jackson ImmunoResearch Laboratories, West Grove, PA, USA) diluted 1:50 in 1× PBS. To avoid nonspecific interactions, the probes were washed five times in 1× PBS for 15 min and then negatively stained with the 2% (w/v) uranyl acetate solution (pH 6.0) for 30 s.

## 4. Conclusions

The coat protein of tobacco mosaic virus served as a carrier for the expression of several peptides [19]. This article describes the first successful example of the efficient systemic expression of the conserved influenza M2e epitope in plants that provided the high-level accumulation of recombinant coat proteins and the efficient assembly of stable rod-shaped M2e-containing chimeric particles in plant leaves. Chimeric TMV-M2e-ser, but not TMV-M2e-cys, virions presented a large amount of foreign peptide exposed on their surface, which retained antigenic specificity and reacted with antibodies against the influenza epitope. Our data are in the same direction as a previous publication describing a cysteine residue in a foreign antigen that affected the assembly of TMV-based viruses [20]. Viral vectors with modified coat proteins cause infections with symptoms that are clearly different from each other and from the wild-type TMV infection. Two point amino acid substitutions (cysteine to serine) in recombinant coat proteins significantly influenced the symptoms and development of viral infections. Our assumption is that the additional serine residues located on the surface of chimeric particles might be phosphorylated and/or glycosylated, and such a modification of viral particles and/or free coat protein subunits could influence the systemic movement of TMV-M2e-ser compared with TMV-M2e-cys. The phosphorylation of coat proteins of plant viruses that affect their replication or movement is not a well-studied topic; only a few examples were published previously [21,22,23,24]. Besides that, cysteine residues in foreign peptide impede the efficient assembly of virus particles with the M2e epitope; immunogold labeling of particles in plant extracts proved that only residual M2e antigens are presented on the surface of virions, but Coomassie staining and western blotting confirmed the efficient systemic expression of recombinant CP-M2e-cys protein (see Figure 3A,B). The rod-shaped geometry of M2e epitopes located on the surface of chimeric TMV-based particles is also different from icosahedral [5] or the helical filamentous [8] arrangement of M2e antigens on the carrier virus-like particles (VLP) described earlier. Some recent examples of the plant-based expression of the M2e epitope [10,11] represent icosahedral carriers (VLPs assembled from HBcAg or L1 protein from human papillomavirus HPV-16) as well. Plant-produced HBcAg-M2e particles induced a protective immune response against one lethal dose (LD_50_) of homologous influenza A infection [11]. Rigid helical TMV-M2e particles proved to be very efficient against challenge with five LD_50_ of either homologous or heterologous (four amino acid changes in the M2e sequence plus cysteine substitutions) influenza A virus [13]. This result looks quite promising, because the protective efficacy of M2e-based vaccines is sensitive to amino acid changes in the target sequence; for example, even a single substitution in M2e led to the appearance of escape mutants of influenza virus [25]. One may suppose that the density and proximity of influenza antigens on the surface of such particle mimics the native tetrameric structure of the M2 protein. 
